# Intensive care discharge summaries for general practice staff: a focus group study

**DOI:** 10.3399/bjgp16X688045

**Published:** 2016-11-22

**Authors:** Suzanne Bench, Jocelyn Cornish, Andreas Xyrichis

**Affiliations:** King’s College London, London.; King’s College London, London.; King’s College London, London.

**Keywords:** critical illness, general practice, patient discharge, qualitative research, rehabilitation

## Abstract

**Background:**

Understanding how patients and relatives can be supported after hospital discharge is a UK research priority. Intensive Care Unit (ICU) discharge summaries are a simple way of providing GPs with the information they require to coordinate ongoing care, but little evidence is available to guide best practice.

**Aim:**

This study aimed at better understanding the information needs of GP staff (GPs and practice nurses) supporting former patients of ICUs and their families following discharge from hospital, and identifying the barriers/facilitators associated with ICU–primary care information transfer.

**Design and setting:**

This was a qualitative exploratory study of practices and participants throughout the UK.

**Method:**

Audiotaped focus group discussions, complemented by small-group/individual interviews, were conducted with 15 former patients of ICUs, four relatives, and 20 GP staff between June and September 2015. Demographic data were captured by questionnaire and qualitative data were thematically analysed.

**Results:**

Findings suggest variability in discharge information experiences and blurred lines of responsibility between hospital and GP staff, and patients/relatives. Continuity of care was affected by delayed or poor communication from the hospital; GPs’ limited contact with patients from critical care; and a lack of knowledge of the effects of critical illness or resources available to ameliorate these difficulties. Time pressures and information technology were, respectively, the most commonly mentioned barrier and facilitator.

**Conclusion:**

Effective rehabilitation after a critical illness requires a coordinated and comprehensive approach, incorporating the provision of well-completed, timely, and relevant ICU–primary care discharge information. Health professionals need an improved understanding of critical illness, and patients and families must be included in all aspects of the information-sharing process.

## INTRODUCTION

Critical illness can have longstanding consequences impacting all aspects of life for patients and their families.^1^^,^^2^ Consequences include fatigue, cognitive impairment, post-traumatic stress disorder, caregiver burden, employment difficulties, and increased health service utilisation, particularly older people or those with significant comorbidities.^1^^–^4 To mitigate these risks, individualised multiprofessional rehabilitation is strongly recommended,^5^^,^^6^ with information sharing being a critical element of this process.^7^^,^^8^

Transfer between secondary and primary care is a particularly high-risk time-point for patients, with avoidable adverse events commonly reported.^9^^,^^10^ Alongside specialist critical care services, GP staff (GPs and practice nurses) have ongoing responsibility for monitoring and managing health following hospital discharge.^6^^,^^11^ Communication across the secondary– primary care interface is, however, often inadequate;^10^^,^^12^^,^^13^ patients discharged home following critical illness report that GPs have little understanding of their needs or those of their families.^14^ Kahn and Angus suggest that this may be because GP staff are unaware of events that occurred in the Intensive Care Unit (ICU).^15^

A lack of information is likely to compromise the ability of GP staff to support patients and families during critical illness rehabilitation. ICU discharge summaries offer a simple solution to this problem. There is, however, a paucity of research on their use, limited to three published questionnaire surveys.^16^^–^18 Collectively, their findings indicate that, although GPs currently receive little information regarding a patient’s in-hospital critical illness experience, they value the information they do receive.

This study explores the views of GP staff, patients, and relatives on ICU discharge information provision.

## METHOD

Using an exploratory qualitative design,^19^ this study aimed at better understanding the information needs of GP staff supporting patients from ICUs and their families after discharge from hospital, and identifying the barriers/facilitators associated with providing ICU–primary care information. The objectives were:

- to assess what information (content and format) about a patient’s critical illness GP practices currently receive, from whom, and how it is received;
- to explore the views of GP staff regarding the information required to support adult patients recovering from a critical illness and their families; and
- to examine patients’ and families’ views about the current and future provision of ICU discharge information to GP staff.

### Selection and description of participants

Using a non-probability purposive sampling approach, former patients admitted to an adult ICU in the UK and subsequently discharged home (and/or a close family member) were recruited by an invitation posted onto the ICUSteps charity website (www.icusteps.org) and posters advertising the study displayed in critical care follow-up clinics in the Greater London area.

**How this fits in**

Hospital discharge is a high-risk time-point for patients recovering from a critical illness. Communication across the secondary–primary care interface is, however, often inadequate. Intensive Care Unit (ICU) discharge summaries offer valuable information if they are well-completed, timely, and relevant to primary care. Staff (that is, mainly GPs and practice nurses) working outside of critical care need an improved understanding of the consequences of critical illness. Patients and families must also be included in all aspects of the information-sharing process.

Initial recruitment of GPs and practice nurses took place from one inner London NHS Clinical Commissioning Group — 08A-Greenwich in South-East London via an e-mail to the practice manager (*n* = 45). E-mail addresses were obtained using publicly-available databases (https://digital.nhs.uk/).

To maximise recruitment, a snowball sampling approach was used^18^ whereby those already recruited identified and encouraged others to participate. Study information was additionally placed on the British Association of Critical Care Nurses (BACCN) website (www.baccn.org) and distributed via national critical care networks. The practices from which GP staff participants were actually recruited varied considerably in terms of registered patients, and the number and type of staff employed (Table 1).

**Table 1.** GP practices: contextual information taken from each practice’s website

**Practice**	**A**	**B**	**C**	**D**
Registered patients, *n*	>24 000	4800	>6000	6500
Catchment	Greenwich, London	Eltham, London	Kent	Bradford
GPs, *n*	14	2	5	4
Nurse practitioners, *n*	–	–	3	1
Nurses, *n*	9	2	3	2
Healthcare assistants, *n*	1	1	1	1

### Data collection

Data were collected from June to September 2015. After providing written consent, participants completed a purposefully designed baseline questionnaire asking for demographic information such as sex, age, and critical illness experience, details about patients’ contact with GP staff after hospital discharge, and hospital information received.

To capitalise on the benefits of group interaction,^20^ 1-hour focus group discussions were used as the primary data collection method. Where it was not possible for people to attend a focus group discussion, they were invited to attend a 30–60 minute semi-structured interview. To maximise participation and mitigate against reported challenges associated with recruiting GP staff,^21^^,^^22^ focus groups and interviews were arranged flexibly to suit the needs of participants. These consisted of face-to-face meetings, telephone discussions, or a mixture of both. In total, three focus group discussions were held with former patients/relatives and three with GP staff. In addition, two small-group (one patient and one GP) and one individual patient interview were conducted (Table 2).

**Table 2.** Sample, setting, and method of data collection

**Participant type**	**Interview type**	**Participants, *n***	**Location of participants**	**Method**
Patients/relatives	Small-group interview 1	2 patients	Leeds	Face-to-face
Patients/relatives	Focus group 1	7 patients 2 relatives	UK wide	Mixed
Patients/relatives	Focus group 2	3 patients 1 relative	Reading	Face-to-face
Patient	Individual interview	1 patient	York	Telephone
Patients/relatives	Focus group 3	2 patients 1 relative	Reading	Face-to-face
GP staff	Focus group 4	5 GPs 2 practice nurses	South-East London	Face-to-face
GP staff	Focus group 5	2 GPs 2 practice nurses	South-East London	Face-to-face
GP staff	Focus group 6	5 GPs 2 practice nurses	Kent	Face-to-face
GP staff	Small-group interview 2	2 GPs	Bradford	Telephone

Focus group discussions were conducted following best-practice guidance.^20^^,^^23^ One researcher facilitated the discussion, while a second non-participant observer noted details of non-verbal communication, contextual issues, and/or the strength of emotional responses. A single researcher facilitated the individual telephone interviews. All focus group discussions/interviews were audiotaped. A topic guide aided data collection; however, participants were also encouraged to explore issues they saw as relevant to the research question.^19^ During focus group research it cannot be guaranteed that all participants will adhere to confidentiality;^20^ however, participants were strongly encouraged not to discuss issues outside of the group. A brief discussion between researchers at the end of each focus group identified potential threats to validity, such as the use of leading questions. Notes of emerging themes were also made, enabling some insight into the extent of data saturation.^24^

### Data analysis

Descriptive statistics (frequencies and percentages) were used to describe the sample and to report the questionnaire data. After transcription, interview data were imported into NVIVO 10. Following the six-stage approach described by Newell and Burnard,^25^ qualitative data were then subjected to a standard process of inductive thematic analysis:

- stage one — notes of key issues after each interview;
- stage two — interview transcripts read and notes made on general themes;
- stage three — transcripts reread and ‘open coded’;
- stage four — overlapping codes amalgamated and reduced (<12);
- stage five — transcripts re-read and text marked with final codes. Codes are compared across transcripts; and
- stage six — coded material forms basis for report.

Analysis was based on full, verbatim transcripts.^20^ Three researchers each coded data from a focus group discussion or interview in which they had not taken part. A second researcher confirmed the first coding before final categories, sub-themes, and themes were determined. A consensus approach was used to resolve any differences in interpretation.

## RESULTS

Fifteen former patients, four family members, and 20 GP staff were recruited. Eight (53%) patients were male and 12 (80%) were aged between 40 and 70 years. All experienced emergency hospital admission and at least 5 days in the ICU. Three of the four relatives (75%) were female, three were spouses, and one was a parent of a patient from an ICU. The 20 GP staff were predominantly aged 25–55 years (*n* = 15; 75%) and 11 (55%) were female. Fourteen (70%) participants were GPs; the others (*n* = 6; 30%) were practice nurses, managers, or nurse practitioners. Eleven (73%) of the 15 patient participants had visited their GP surgery more than 10 times since hospital discharge. Twelve (80%) indicated that this was because of a problem related to critical illness, yet almost half (*n* = 7; 47%) believed that their GP had not received any ICU discharge information; either directly or embedded within a standard hospital discharge notification. GP staff corroborated this view with eight (40%) participants reporting that they only occasionally received discharge information providing any detail about a patient’s stay in the ICU.

An initial 170 codes were amalgamated to produce three key themes, underpinned by a number of sub-themes and lower-level categories (Box 1).

Box 1. **Themes, sub-themes, and categories**

**Table:** 

**A coordinated and comprehensive approach**			**A need to know**			**Barriers and facilitators**	
		
**Ongoing support**	**Blurred lines of responsibility**	**Cross-boundary working**	**Health status**	**Forward planning**	**Services**	**Structure/process/resource**	**Knowledge/experience/understanding**
Duration of hospital stay Changing status Physical effects Psychosocial effects	Patient/family initiated Importance of relatives GP coordinator of care Wider responsibility	Transfer of care communication Variations in care provision Discharge planning	Physical and psychological Need to understand Medications	Checklists and instructions Knowing what to expect Self-management	Follow-up and support services Psychological support	Timely and appropriate Detail versus succinct Lay language Routine follow-up No information Technology Time and money Diaries	Need for flags Junior staff Focus on physical health Education and exposure

### A coordinated and comprehensive approach

A range of physical and psychosocial consequences of critical illness was described, with patients and relatives strongly emphasising the need for ongoing support following hospital discharge:

‘*They should realise that if you’ve been in ICU, no matter how long … you do suffer, whatever happens. And I think it’s got to be portrayed to the GPs.*’

(Patient formerly in an ICU, focus group 1)

Participants described the fluctuating nature of the recovery process and emphasised the need for information to be delivered as part of a coordinated comprehensive approach, from the ICU to the ward, through to primary care. Although some patients and relatives reported receiving really good support after discharge, others described it as a process of luck:

‘*I had a great GP but there’s other GPs in the practice who didn’t understand at all … I was lucky. And I don’t think it should be a lottery, really, because it’s not your fault you end up in ICU.’*

(Patient formerly in an ICU, focus group 1)

The concept of ‘blurred lines of responsibility’ was prominent, with varying views regarding who held the responsibility for providing follow-on care. An excerpt from one of the GP focus group discussions highlights that follow-up is not routine and that some GPs see their role as responding to patients’ requests for help:

G1: ‘There’s a curious assumption in hospitals that GPs will call people in and sort them out … And we think well, no! We respond to the patients coming to us.’

G2 ‘I think there might be a variation, so if somebody had had a significant illness and you knew them, you might get someone to ring them and ask them if they’d like to come in and see you. I think there’d probably be a variation but there’s no routine; there wouldn’t be a routine “you must come and see me because you’ve been in hospital”.’

I: ‘So it is quite possible that a patient could be discharged from hospital and then if they were doing OK and felt they were doing OK, that they wouldn’t have any contact with you afterwards?’

G2: ‘Absolutely.’

G1: *‘Very possible, yes.*’ (All GPs, focus group 4)

There was agreement that the responsibility for sharing information about the hospital stay is often left to the patient and/or a family member:

‘*It’s usually the patient who gives us a bit of a summary as to what’s happened because the discharge summary does lag a bit sometimes. Usually the patients are quite on the ball, which is good.*’

(GP, focus group 6)

Family members were also seen to have a key role to play in facilitating successful transition from hospital to home. Concerns were expressed, however, about those who did not have anyone to take on this role:

‘*My wife if she was here, she would say in some ways I was a burden because she hadn’t been given all the facts, somebody just gave her a big parcel of 134 tablets … why is he taking this tablet, why, what, there was nothing.*’

(Patient formerly in an ICU, small-group interview 1)

‘*My brother was on the case, my other half was on the case, my parents were on the case … what happens if I didn’t have someone with me?*’

(Patient formerly in an ICU, focus group 3)

The importance of effective transfer of care communication across organisational boundaries was highlighted. Participants discussed how ICU discharge information might be aligned with standard hospital discharge notifications:

‘*I’m just wondering in my mind how useful an ITU discharge summary would be for the GP or whether it would be sufficient within the final discharge itself just to mention the patient spent a period on ITU.*’

(GP, focus group 4)

‘*I think discharge documents should commence when you go into hospital so that the treatment can be summarised with the drugs and physio, etc. So it’s an ongoing live document that maybe runs to two pages when you’re discharged.*’

(Patient formerly in an ICU, focus group 3)

### A need to know

There were varied opinions about the information required by different individuals, but agreement that GP staff need information about key ICU events and potential psychological as well as physical health consequences:

‘*I think the GP should also know about any interventions and problems that I might have had before that cardiac arrest … And the reason why I say is because it all affects the subsequent recovery … If they’re going to be able to help me with the delirium that I suffered they need to know how serious the delirium was; it varies enormously. And I think they need to know about any anxiety, depression, and my mental state generally … And the physical abilities and limitations as well … They need this information to give good follow-up service and if my own GP can help me out, that’s the summary of it all.*’

(Patient formerly in an ICU, focus group 1)

‘*We tend not to get much information about psychosocial functioning or problems and even the nursing notes can sometimes be, “Medication given as prescribed”, which isn’t terribly helpful, so we can obviously experience patients who come and it has been a pretty distressing event for them but we aren’t forewarned.*’

(GP, focus group 6)

Knowing what happened in the ICU helped GPs prepare for a consultation with the patient. One GP said:

‘*I think some sort of brief summary sent to the GP is actually quite useful; keeps us in the loop, we know what’s going on, and we’re sort of prepared when they turn up. It is embarrassing to see someone who’s had a serious illness and you haven’t got a clue what’s happened.*’

(GP, focus group 4)

The need to help patients better understand what happened in the ICU was also evident:

‘*It would be lovely if that bottom bit was a narrative that the patient could understand about what had happened to them whilst they were in ICU.’*

(GP, small-group interview 2)

*‘I knew I had hallucinations and I did go back to intensive care with my family because I couldn’t work out if the people that I remembered as being in there were real or not. I had no idea if they were real or not. So if someone had given me a full copy of the discharge list … My GP can explain it to me.*’

(Patient formerly in an ICU, focus group 3)

In addition, there was agreement that GPs and patients need a rationale for decisions made in the hospital. This was considered particularly true in relation to the ongoing plan for medication management:

G1: ‘The medication on any discharge summary and knowing why they’ve been stopped or why they’ve been started …’

G2: ‘Yeah, it would be really lovely. The timeline for stopping medications … would be amazing.’

G1: *‘That would be great because GPs are known to be really bad at stopping things.*’ (Both GPs, small-group interview 2)

There was also consensus that ICU discharge information should be forward thinking, alerting GPs to what had already been done, what needed to be done, and things to look out for after hospital discharge; similar to a checklist. One GP commented:

‘*It would be knowing what’s happened and what to look out for, so saying, “This person has had an extended stay on ICU. They are at risk of post-traumatic stress” and to be aware of that or whatever, or “They’ve had a tracheostomy and 20% of people get tracheal stenosis. The things to look out for are this, this and this*”.’

(GP, small-group interview 2)

Instructions were seen as important, for GP staff in terms of follow-up requirements, but also for the patient and family members to aid self-management:

‘*There were no instructions on how to look after a person you’ve just taken home from hospital or anything like that. You just had to work it out as you went along.*’

(Relative of a patient formerly in an ICU, focus group 1)

‘*We wanted to be able to do it ourselves. We’re quite capable of doing it if we’re told what we need.*’

(Patient formerly in an ICU, focus group 1)

Information for GPs about specific support services available for patients requiring critical care was also seen as important, particularly with regards to providing psychological support. Participants talked about the need for referral to services such as talking therapies, cognitive behavioural therapy, support groups, and trauma and bereavement counselling that were relevant to ICU.

Other common requirements included physiotherapy, dietetic advice, and voice and wound management. Other than to manage wounds, participants did not envisage a specific role for practice nurses in supporting patients after a critical illness.

### Barriers and facilitators

All participants discussed the need for GP staff to have a better understanding about the consequences of critical illness:

‘*I don’t know enough about complications of things that come out of ICU … There are probably medical things that post-ICU people could benefit from that I just don’t know about … if they come they might have had an admission 6 months before, they had one delirium episode and they come with raised anxiety. I wouldn’t know to link those two things.*’

(GP, focus group 5)

This lack of understanding was also considered a problem for many ward staff:

‘*The ward needs details … they are not intensive care practitioners and they do not know the details. So they can’t really complete the detailed discharge note for your time in intensive care.*’

(Patient formerly in an ICU, focus group 3)

The fact that junior doctors, with little experience of ICU, often write discharge notifications was seen as a key barrier to effective transfer of care communication:

‘*The people who write them are always the most junior members of the team … and I think that’s why in the normal hospital, ward discharge summaries after they’ve been to ITU will just say “been to ITU” because they probably can’t understand what’s happened themselves so they wouldn’t go into the details there. And that’s the nature of medicine; it’s always the most junior who does it.’*

(GP, focus group 4)

In contrast, the use of lay language was considered to be a means of facilitating more effective understanding for everyone:

‘*I couldn’t make head or tail of what the discharge note said or what it meant. So it would have been nice to have had a summary sheet in plain language that a supporter or carer could have dealt with instead of having to look stuff up on the internet.*’

(Patient formerly in an ICU, focus group 1)

Exposure to patients who had experienced critical illness was an infrequent occurrence for GPs, who described difficulties identifying those requiring support:

*‘… so he sees one a month. I’ve been in this job for just over a year and I can remember three patients — adult patients; paediatrics perhaps, a bit more but that’s how much we would receive.*’

(GP, focus group 6)

‘*Unfortunately, they don’t stand out. They’re just one of several people who’ve had a traumatic experience with long-term consequences.*’

(GP, focus group 5)

The current structure and process of discharge information provision was identified as a further barrier, with participants describing a lack of information and emphasising the need for information to be relevant and timely:

‘*There was nothing from intensive care to my GP to say what had happened to me, what drugs they’d given me. Absolutely nothing at all.*’

(Patient formerly in an ICU, focus group 3)

‘*I guess it is this thing about the speed of when these letters come in, and if they’re going to need support it’s probably going to be early on. So if it takes 2 or 3 weeks or something for a letter to come through and we don’t know by then, I think it is the speed of things getting to us.*’

(GP, focus group 4)

Technologies such as computers, telephone, Skype, and e-mail were identified as potential facilitators to improve the timeliness of information transfer and allow for a system that could flag vulnerable patients:

P1: ‘*Flags should be up shouldn’t they for people …’*

P2: *‘Especially being in CCU etc. etc.*’ (Former ICU patients, small-group interview 1)

The need for systems to be compatible, however, across organisation boundaries and healthcare providers was also highlighted.

Finally, information itself was described as both a barrier and facilitator. A tension between having enough information to support effective decision-making and being bombarded with an overwhelming amount of information was described:

‘*Luckily for me the discharge note was very, very detailed, really, really good.*’

(Patient formerly in an ICU, focus group 1)

‘*Sometimes ICU discharge summaries if you do see them are quite detailed and it’s quite difficult to go through and pick out the relevant details. Which obviously ICU is a very detailed place.*’

(GP, focus group 4)

GPs’ desire for more succinct information and current system failures were, in part, attributed to resource constraints:

‘*I want to provide holistic care … but the reality is that I wouldn’t have the time to do that and that feels like another thing … I would tend to get a batch of 40 or 50 in as quick a time as possible and I tend to try and get them done in 45 seconds per discharge.*’

(GP, focus group 5)

## DISCUSSION

### Summary

Effective critical care rehabilitation requires a coordinated and comprehensive approach, a responsibility shared between in-hospital and primary care teams. Staff outside of critical care need an improved understanding of critical illness, and patients and families must be included in all aspects of the information-sharing process. The provision of well-completed, timely, and relevant ICU–primary care discharge information may improve communication across organisational boundaries and enhance a patient’s critical illness recovery experience.

### Strengths and limitations

This first exploratory study presents a set of rich and insightful data reflecting service users’ and service providers’ views. All participants self-selected, however, and all patients and family members were white British. GPs also spoke more than other health professionals during the focus group discussions. The views of ethnic minority groups and other primary care practitioners may, therefore, not be fully reflected. Despite the small, self-selected sample, there was evidence of data saturation, with similar recurring issues identified in many of the transcripts. Rigour was also maintained by cross-checking of codes and themes by researchers against original transcripts to ensure that findings could be substantiated.

### Comparison with existing literature

The present findings support the value of ICU discharge information, while highlighting that its provision to GP staff is rare. GPs (*n* = 36) sent ICU discharge summaries in one NHS Trust in England agreed that they were helpful (69%) and wanted the practice to continue (86%).^16^ Wong and Wickham report, however, that only 22 (36.7%) UK ICUs sent a discharge letter to the patient’s GP, with significant variation in the information provided.^17^ Similarly, a lack of discharge information was identified as one of five independent factors associated with French GPs’ (*n* = 1561) dissatisfaction with ICU staff (odds ratio [OR] 3.39 [1.70 to 6.76]).^18^

In line with the experiences of the present study participants, research indicates that hospital discharge summaries are poorly completed and often delayed.^10^^,^^12^^,^^13^^,^^26^^–^28 Since October 2015, NHS England has required all transfer of care communications to be electronic.^29^ This, alongside better coding systems, should improve the delays described and highlight individuals most at risk. The present findings suggest, however, that the IT infrastructure is not yet providing efficient communication across organisational boundaries.

There are similarities between the present data and the information that patients discharged from other in-hospital care facilities consider to be important.^10^ Examples are information about followup arrangements already in place and instructions for the patient and GP staff. The present data suggest, however, that GPs supporting patients recovering from critical illness need, in addition, information about what happened in the ICU. There also needs to be a more balanced emphasis on managing both mental and physical health, a view endorsed by the Department of Health.^30^^,^^31^ The important role taken on by relatives in coordinating the transition from hospital to home, and the impact that this can have,^3^ also needs to be recognised and supported.

The present findings concur with those of Cresswell *et al* that junior doctors write most hospital discharge notifications, despite receiving little formal teaching on how to do this.^12^ It was also found that GPs had little understanding of critical illness. Educational input that is commenced during initial training, and which is sustained through continuing professional development,^12^^,^^32^ is required to optimise this area of practice for all healthcare professionals.

This study highlights the need for a comprehensive and coordinated approach to transfer of care communication. Work to improve ICU to ward discharge summaries demonstrates some progress.^33^ A single ongoing document where all members of the multidisciplinary team can add information at each transition point might help achieve aspirations for streamlined care.^13^^,^^27^^,^^31^ Findings from the current study and previous work by Bench *et al* further suggest that the use of lay language and giving ICU discharge summaries to patients facilitates a common understanding of the critical-illness experience and shared decision-making during recovery.^34^

### Implications for research and practice

Critical care rehabilitation, as recommended by the National Institute for Health and Care Excellence (NICE),^6^ requires follow-up provided by specialist teams and support from other in-hospital and primary care staff. Effective communication between healthcare professionals is a key element of this shared-care approach. A hospital discharge notification template produced by the Royal College of Physicians is currently recommended for all transfer-of-care communications.^35^

To improve the ability of GP staff to support patients recovering from critical illness, drawn from the present findings is a proposal to develop an additional template specifically for ICU discharge summary information and to consider how best to embed ICU discharge information into existing systems and processes. Given the challenges reported by the present study participants, future research is suggested to systematically examine organisational and system-level barriers to the implementation of such a discharge summary as well as related resource implications.
